# Role of Hydraulic Signal and ABA in Decrease of Leaf Stomatal and Mesophyll Conductance in Soil Drought-Stressed Tomato

**DOI:** 10.3389/fpls.2021.653186

**Published:** 2021-04-29

**Authors:** Shuang Li, Junming Liu, Hao Liu, Rangjian Qiu, Yang Gao, Aiwang Duan

**Affiliations:** ^1^Key Laboratory of Crop Water Use and Regulation, Ministry of Agriculture and Rural Affairs, Farmland Irrigation Research Institute, Chinese Academy of Agricultural Sciences, Xinxiang, China; ^2^Graduate School of Chinese Academy of Agricultural Sciences, Beijing, China; ^3^School of Applied Meteorology, Nanjing University of Information Science and Technology, Nanjing, China

**Keywords:** drought, leaf water potential, abscisic acid, stomatal conductance, mesophyll conductance, intrinsic water use efficiency

## Abstract

Drought reduces leaf stomatal conductance (g_s_) and mesophyll conductance (g_m_). Both hydraulic signals and chemical signals (mainly abscisic acid, ABA) are involved in regulating g_s_. However, it remains unclear what role the endogenous ABA plays in g_m_ under decreasing soil moisture. In this study, the responses of g_s_ and g_m_ to ABA were investigated under progressive soil drying conditions and their impacts on net photosynthesis (A_n_) and intrinsic water use efficiency (WUE_i_) were also analyzed. Experimental tomato plants were cultivated in pots in an environment-controlled greenhouse. Reductions of g_s_ and g_m_ induced a 68–78% decline of A_n_ under drought conditions. While soil water potential (Ψ_soil_) was over −1.01 MPa, g_s_ reduced as leaf water potential (Ψ_leaf_) decreased, but ABA and g_m_ kept unchanged, which indicating g_s_ was more sensitive to drought than g_m_. During Ψ_soil_ reduction from −1.01 to −1.44 MPa, Ψ_leaf_ still kept decreasing, and both g_s_ and g_m_ decreased concurrently following to the sustained increases of ABA content in shoot sap. The g_m_ was positively correlated to g_s_ during a drying process. Compared to g_s_ or g_m_, WUE_i_ was strongly correlated with g_m_/g_s_. WUE_i_ improved within Ψ_soil_ range between −0.83 and −1.15 MPa. In summary, g_s_ showed a higher sensitivity to drought than g_m_. Under moderate and severe drought at Ψ_soil_ ≤ −1.01 MPa, furthermore from hydraulic signals, ABA was also involved in this co-ordination reductions of g_s_ and g_m_ and thereby regulated A_n_ and WUE_i_.

## Introduction

Soil water scarcity is one of the major environmental constraints to the plant physiological processes and yield (Easlon and Richards, 2009; Olsovska et al., 2016). To achieve high plant water-use efficiency under a drier environment in the future, it is essential to improve crop photosynthesis and productivity with a given unit of water (Flexas et al., 2013). For C_3_ plants, leaf photosynthesis is strongly limited by three factors, i.e., stomatal conductance (g_s_), mesophyll diffusion conductance to CO_2_ (g_m_), and biochemical photosynthetic capacity (Grassi and Magnani, 2005; Cano et al., 2013). g_s_ and g_m_ determine the diffusion of CO_2_ from ambient air of leaf to sub-stomatal cavities and from the sub-stomatal cavities to chloroplast stroma, respectively (Flexas et al., 2002; Niinemets et al., 2009). Recent studies have shown that both g_s_ and g_m_ were the main limitations for maximum photosynthesis under drought conditions (Tosens et al., 2016; Wang et al., 2018). Therefore, revealing the mechanisms underlying the decreases of g_s_ and g_m_ in response to drought is necessary for enhancing our understanding of plant adaptation to water limitation.

Different regulatory mechanisms such as chemical messengers like abscisic acid (ABA), electrical signals, and hydraulic signals have been identified in the control of stomatal movement (Dodd, 2005; Ache et al., 2010; Tombesi et al., 2015; Huber et al., 2019). Despite the large list of candidates in regulating guard cells, ABA and hydraulic signals have gained most of the attention in regulating stomatal aperture. ABA is a phytohormone that has been involved in different strategies of plants to avoid excessive water loss, and many reports demonstrated its important role in stomatal control (Wilkinson and Davies, 2002; Assmann and Jegla, 2016). The decrease of g_s_ in response to drought has been generally modulated by the accumulation of leaf ABA in a wide number of plant species including soybean, grapevine and tomato (Liu et al., 2005; Tombesi et al., 2015; Yan et al., 2017). However, stomata closed with a wide range of variations of leaf hydraulic signals, such as leaf water potential (Ψ_leaf_), possibly due to differences of experimental plant materials and the intensity of applied drought under investigation. For example, g_s_ decreased with decreasing Ψ_leaf_ during leaf dehydration (Kim et al., 2012; Wang et al., 2018). On the contrary, other studies showed that stomata closed with little change in Ψ_leaf_ under moderate soil drying, but both parameters decreased under severe drought (Tardieu, 1998; Yan et al., 2017), or g_s_ decreased as Ψ_leaf_ increased under mild soil drying but then no significant relationship existed between both variables with continued soil drying (Kudoyarova et al., 2007). It is difficult to explore the response of Ψ_leaf_ and g_s_ under a single soil water condition. Progressive soil drying, representing a natural process of soil water loss, could help us explore the dynamic responses of g_s_ to Ψ_leaf_ during drying process.

Leaf mesophyll conductance to CO_2_ (g_m_) has been recognized to be finite, variable, and rapid acclimation to varying environmental conditions. Although a reduction in g_m_ response to soil drought has been reported in many studies, the mechanisms underlying this reduction have not been elucidated substantially (Flexas et al., 2002; Théroux-Rancourt et al., 2014; Sorrentino et al., 2016). Recent studies on hydraulic signals suggested that the parallel decreases in g_s_ and g_m_ were caused by leaf hydraulic vulnerability as a result of decrease in Ψ_leaf_ (Wang et al., 2018). Similarly, g_m_ was strongly correlated with leaf hydraulic conductance (K_leaf_), as the ratio of transpiration rate to the water potential driving force across the leaf (K_leaf_ = transpiration/ΔΨ_leaf_), across species under light-saturated conditions (Xiong et al., 2018). This correlation between g_m_ and leaf hydraulic signals might be due to CO_2_ partially shared common diffusion pathways with H_2_O through mesophyll tissues (Ferrio et al., 2012). These studies confirmed that leaf hydraulic signals played an essential role in controlling g_m_ in response to drought. However, the effects of chemical ABA signal on g_m_ are not consistent. Vrabl et al. (2009) did not observe any reduction in g_m_ when applied exogenous ABA in *Helianthus annuus* plants. In line with this, Flexas et al. (2013) found that g_m_ was highly insensitive to endogenous ABA among ABA-insensitive and ABA-hypersensitive genotypes or to exogenous ABA application in *Arabidopsis thaliana*. However, several studies yielded contrasting results. For instance, Mizokami et al. (2015) compared the responses of g_m_ to leaf ABA in wild type and ABA-deficient mutant of *Nicotiana plumbaginifolia* and confirmed that the increase in leaf ABA concentration was crucial for the decrease in g_m_ under drought conditions. Still, g_m_ reduced effectively in response to ABA in a short term in three of the four species in Sorrentino et al. (2016). Recently, Mizokami et al. (2018) examined the responses of g_m_ to high CO_2_ and ABA application and revealed that g_m_ was able to respond to high ABA levels, which was intrinsically different from the response to the elevated CO_2_. These contrasting results possibly due to species differences or the experimental approaches utilized to modify ABA, e.g., the exogenous ABA concentration or the applying period. In brief, it has been largely demonstrated that hydraulic signals play an important role in regulating g_m_, while the role of ABA on g_m_ is still not unequivocal. Therefore, a deep understanding about the mechanisms of g_m_ response to endogenous ABA under progressive soil drying conditions awaits further investigation.

Leaf intrinsic water use efficiency (WUE_i_), expressed as the ratio of net photosynthetic rate (A_n_) to g_s_ at leaf level, can explain instantaneous responses to environmental factors (Flexas et al., 2016; Qiu et al., 2019). Improving WUE_i_ need increase A_n_ and decrease g_s_ simultaneously. Using A_n_/g_s_ to explain the changes of WUE_i_ would be too coarse due to the decrease in g_s_ inevitably affect CO_2_ uptake and thereby limit A_n_. g_m_ determines the CO_2_ concentration at the carboxylation site in the chloroplast, increasing g_m_ would increase A_n_ without increasing water loss. Therefore, g_m_ might play a role in improving WUE_i_. Despite all of the negative impacts of drought stress on leaf gas exchange, many studies reported that drought was beneficial to improve WUE_i_ (Liu et al., 2005; Xue et al., 2016). However, the reasons of this improvement of WUE_i_ have not been elucidated clearly. Evidences have suggested that g_m_/g_s_ played a key role on increasing WUE_i_ in response to water limitation (Flexas et al., 2016; Han et al., 2016). Revealing the exact responses of g_m_/g_s_ or WUE_i_ to stressed signals especially ABA under progressive soil drought would be of great interest in the selection of varieties with high yield in breeding and strong adaptability under varied environmental conditions.

In this study, relationships between g_s_, g_m_, and Ψ_leaf_ or ABA were examined in tomato seedlings under progressive soil drying conditions. The objectives of this study were (i) to evaluate the effects of limiting factors of g_s_ and g_m_ on A_n_ in tomato plants during progressive soil drying, (ii) to investigate the responses of g_s_ and g_m_ to drought signals (Ψ_leaf_ and ABA) under increasing drought stress, and (iii) to reveal the effects of g_s_/g_m_ on WUE_i_ in tomato seedlings during the progressive soil drying.

## Materials and Methods

### Plant Material and Soil Water Treatments

Seeds of tomato (*Solanum lycopersicum* L., cv. Helan108) were sown in nursery seedling plate with substrate (sphagnum peat, Pindstrup Mosebrug A/S, Ryomgaard, Denmark). When the second true leaf emerged, tomato seedlings were transplanted into 5.3 L pots (height 30 cm, diameter 15 cm). Each pot was filled with 6.5 kg air-dried sandy loam soil. The gravimetric field water capacity (θ_FC_) and wilting point were 22% (g g^−1^) and 6.8% (g g^−1^), respectively. After transplanting, all pots were irrigated to 85% θ_FC_ with Hoagland solution [5 mM KNO_3_, 5 mM Ca (NO_3_)_2_ 4H_2_O, 1 mM KH_2_PO_4_, and 1 mM MgSO_4_ 7H_2_O, 1 ml l^−1^ micronutrients, pH = 6.0]. Seedlings were cultivated in an environment-controlled chamber [day/night air temperature 25/18°C, 50–60% relative humidity, 12 h photoperiod at 600 μmol m^−2^ s^−1^ photosynthetic photon flux density (PPFD) supplied by LED lamps from 7:00 to 19:00]. All pots were weighted daily at 8:00 a.m. to calculate daily irrigation amount. During the experiment, same volume of Hoagland solution was applied to all pots to avoid nutrient differences. Soil water content was expressed as relative soil water content (RSWC), i.e., the ratio between the current soil moisture (θ_C_) and θ_FC_.

Water treatments (including well-watered and progressive drought-stressed treatments) were conducted at the 27 day after transplanting (DAT). For the well-watered treatment, RSWC was maintained within the range of 70–82% θ_FC_ throughout the experiment. Plants remained well-watered acted as a control group (CK). For the drought-stressed treatment (withholding water), RSWC decreased from 82.90% θ_FC_ to 37.27% θ_FC_ from 27 to 33 DAT. On each day of the drying period (28–33 DAT), the relevant experimental indexes were measured and collected for the two treatments.

### Leaf Gas Exchange and Chlorophyll Fluorescence Measurements

Leaf gas exchange and chlorophyll fluorescence were measured simultaneously using an open gas exchange system Li-Cor 6400 photosynthesis system (Li-Cor Inc., Lincoln, NE, USA) equipped with an integrated leaf fluorometer chamber (Li-Cor 6400-40) from 9:00 to 14:00 h. All measurements were recorded on the same fully expanded leaves (the 6th or 7th leaves from the base of the plant) during 28–33 DAT, using two or six replicate plants for CK and water stressed treatment, respectively. During the measurements, the PPFD was kept at 1500 μmol m^−2^ s^−1^, the sample CO_2_ concentration was maintained at 400 μmol mol^−1^ with a CO_2_ cylinder. Relative humidity was kept at 55%. Leaf gas exchange, chlorophyll fluorescence and leaf temperature were recorded when A_n_ was stabilized on these conditions (usually 20 min after clamping the leaf). After that, A-C_i_ response curves were conducted. During the measurements, the PPFD was kept as constant of 1500 μmol m^−2^ s^−1^, sample CO_2_ concentration was adjusted in a series of: 400, 300, 200, 150, 100, 50, 400, 400, 600, 800, 1000, 1200, 1400, 1600 μmol mol^−1^.

The intrinsic water use efficiency (WUE_i_, μmol CO_2_ mol^−1^ H_2_O) was calculated as the ratio of net photosynthetic rate divided by stomatal conductance:

(1) $$\begin{array}{l} WUE_{i}=A_{n}/g_{s} \end{array}$$

where A_n_ is net photosynthesis rate (μmol CO_2_ m^−2^ s^−1^), g_s_ is stomatal conductance (mol H_2_O m^−2^ s^−1^).

The actual photochemical efficiency of photosystem II (Φ_*PSII*_) was determined by measuring steady-state fluorescence (F_s_) and maximum fluorescence (${\text{F}}_{\text{m}}^{\prime }$) during a light-saturating pulse of ca. 8000 mmol m^−2^ s^−1^:

(2) $$\begin{array}{l} \Phi_{PSII}=(F_{m}\prime -F_{s})/F_{m}\prime \end{array}$$

The electron transport rate (*J*_f_) was then calculated as:

(3) $$\begin{array}{l} J_{f}=\Phi_{PSII}\times PPFD\times \alpha \times \beta \end{array}$$

where PPFD was maintained at 1500 μmol m^−2^ s^−1^ on both the well-watered and water-stressed leaves. α represents the leaf absorptance and β reflects the partitioning of absorbed quanta between photosystems II and I. α and β were assumed to be 0.84 and 0.5 in the study, respectively (Laisk and Loreto, 1996; Flexas et al., 2002).

### Estimation of g_m_ by Gas Exchange and Chlorophyll Fluorescence Method

g_m_ was calculated by the variable J method of Harley et al. (1992), as follows:

(4) $$\begin{array}{l} g_{m}=\frac{A_{n}}{C_{i}-\frac{\Gamma^{*}\left(J_{f}+8\left(A_{n}+R_{d}\right)\right)}{J_{f}-4\left(A_{n}+R_{d}\right)}} \end{array}$$

where C_i_ represents intercellular CO_2_ concentration (μmol CO_2_ mol^−1^), R_d_ represents the light mitochondrial respiration (μmol CO_2_ mol^−1^), which was calculated as 1/2 of the dark respiration Xiong et al. (2018), Γ^*^ is the chloroplast CO_2_ compensation point (μmol CO_2_ mol^−1^), a leaf temperature-dependent parameter, and estimated as:

(5) $$\begin{array}{l} \text{Parameter}=\exp\left(c-\frac{\Delta H_{a}}{R\cdot T_{K}}\right) \end{array}$$

where *c* is the scaling constant (dimensionless), *H*_*a*_ is the energies of activation (KJ mol^−1^), and R is the molar gas constant (8.314 J K^−1^ mol^−1^). At the leaf temperature of 25°C, *c* and *H*_*a*_ in *S. lycopersicum* were equal to 12.7 and 23.2 (KJ mol^−1^), respectively (Hermida-Carrera et al., 2016). T_k_ is the leaf absolute assay temperature (K), which was recorded by the LI-6400 system and corrected to Kelvin temperature.

Given the potential errors in estimation made by the variable J method, sensitivity analyses were conducted to determine the effect of ±20% error of R_d_, Γ^*^, J_f_, and C_i_ on calculation of g_m_.

### Photosynthetic Limitation Analysis

The relative photosynthesis limitations of A_n_ resulting from g_s_ (l_s_), g_m_ (l_m_), and biochemical photosynthetic capacity (l_b_) (l_s_ + l_m_ + l_b_ = 1) was determined using the method of Grassi and Magnani (2005), as follows:

(6) $$\begin{array}{l} l_{s}=\frac{g_{t}/g_{sc}\cdot \partial A/\partial C_{c}}{g_{t}+\partial A/\partial C_{c}} \end{array}$$

(7) $$\begin{array}{l} l_{m}=\frac{g_{t}/g_{m}\cdot \partial A/\partial C_{c}}{g_{t}+\partial A/\partial C_{c}} \end{array}$$

(8) $$\begin{array}{l} l_{b}=\frac{g_{t}}{g_{t}+\partial A/\partial C_{c}} \end{array}$$

where g_sc_ is the stomatal conductance to CO_2_ (mol CO_2_ m^−2^ s^−1^), g_sc_ = g_s_/1.6, g_t_ is the total conductance to CO_2_ from the leaf surface CO_2_ to chloroplast (1/g_t_ = 1/g_sc_ + 1/g_m_). According to the Farquhar model (Farquhar, 1980), ∂A/∂C_c_ can be calculated as follows:

(9) $$\begin{array}{l} \partial A/\partial C_{c}=\frac{V_{c\text{ max}}\cdot \left(\Gamma^{*}\text{+}{\text{K}}_{\text{c}}\left(1+O/K_{o}\right)\right)}{{\left(C_{c}+K_{c}\cdot \left(1+O/K_{o}\right)\right)}^{2}} \end{array}$$

where *K*_c_ and *K*_o_ are the Rubisco Michaelis–Menten constants for CO_2_ and O_2_, both of them were temperature-dependent and calculated as Equation (5). Specific values of these parameters in Equation (5) were obtained from Sharkey et al. (2007). *O* is the atmospheric O_2_ concentration (210 mmol mol^−1^). V_cmax_ is the maximum carboxylation capacity (μmol m^−2^ s^−1^). V_cmax_ was calculated from the A/C_i_ curve fitting method (Long and Bernacchi, 2003).

### Soil and Leaf Water Potential Measurement and Shoot Sap Collection

Leaf water potential (Ψ_leaf_) was measured on the same leaves as the measurement of gas exchange. Soil samples at the 10–12 cm under soil surface were collected to measure soil water potential (Ψ_soil_). Both Ψ_leaf_ and Ψ_soil_ were measured by the WP4C Dewpoint Potentiometer (Meter Group Inc., Pullman, WA, USA) with two or six repetitions for CK and water stressed treatment. Meanwhile, the shoot part (including stem and leaf) was put into the Model 3115 pressure chamber (Plant Moisture Equipment, Santa Barbara, CA, USA). Pressure was increased gradually until sap outflowed at the cut surface. After discarding the first 1–2 drops, nearly 2 ml of sap was collected into centrifuge tube frozen in liquid nitrogen and then stored at −80°C for ABA analysis.

### ABA Determination

The concentration of ABA was determined as previously described by Li et al. (2020). Briefly, sap ABA concentration was measured with a high-performance liquid chromatography-tandem mass spectrometry (Agilent Technologies Inc., Santa Clara, CA, USA), quantitated as the methods of isotope internal standard.

### Statistical Analysis

All statistical analyses were performed using SPSS 16.0 (IBM Corp., Armonk, NY, United States). The significance of differences between mean values was assessed by One-way analysis of variance (ANOVA) according to Dennett's test at *P* < 0.05 level. Regressions were fitted by linear or non-linear models, and the model with higher regression coefficient (*r*^2^) was selected. Regression lines was shown when *P* < 0.05. All graphics and regressions were performed in Origin-Pro 2017 (Origin Lab, Northampton, MA, USA).

## Results

### Dynamic of Soil Water Status

**Figure 1:**
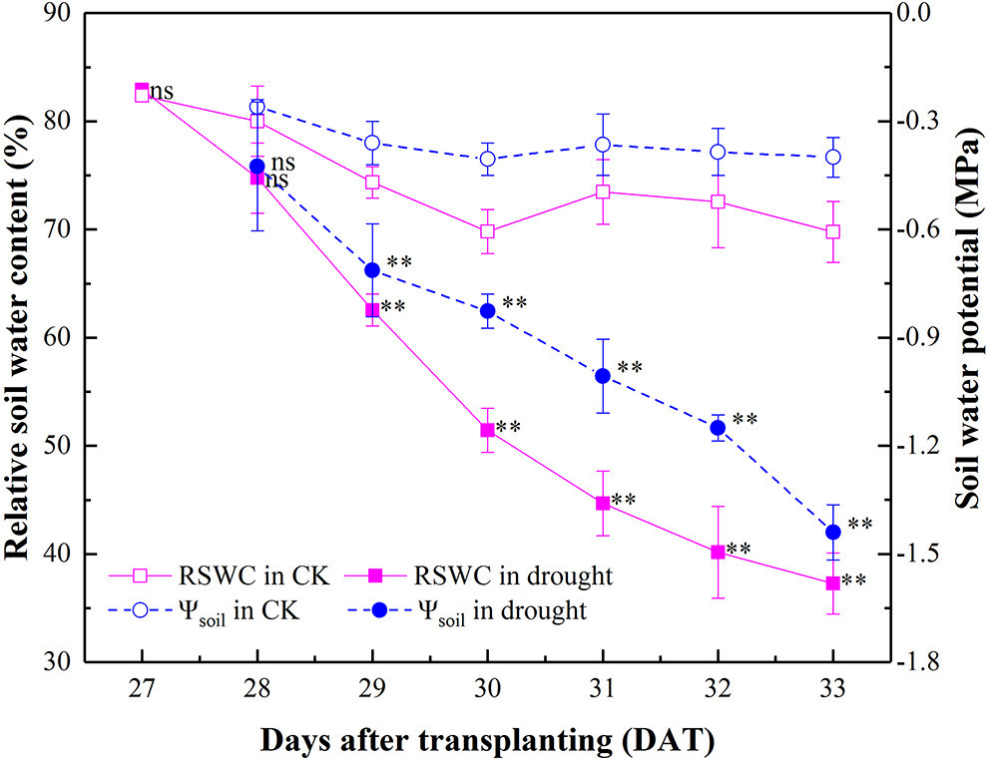
Dynamics of RSWC and Ψ_soil_ in the well-watered (CK) and drought-stressed tomato seedlings during 27–33 DAT. Mean values and SD were presented (*n* = 6). ns indicated no significant difference and ^**^ indicated significant difference at *P* < 0.01 level between drought and well-watered treatment.

Relative soil water content (RSWC) and Ψ_soil_ of the well-watered pots were maintained at an average of 75.13% and −0.43 MPa, indicating no water stress occurred during the experiment. By withholding irrigation from 27 to 33 DAT during the progressive drying process, RSWC in the drought treatment decreased gradually from 82.90 to 37.27% and Ψ_soil_ decreased by 1.04 MPa correspondingly. Interestingly, significant reduction of both RSWC and Ψ_soil_ occurred simultaneously at 29 DAT (Figure 1).

### Effects of Drought on Ψ_leaf_ and ABA

**Figure 2:**
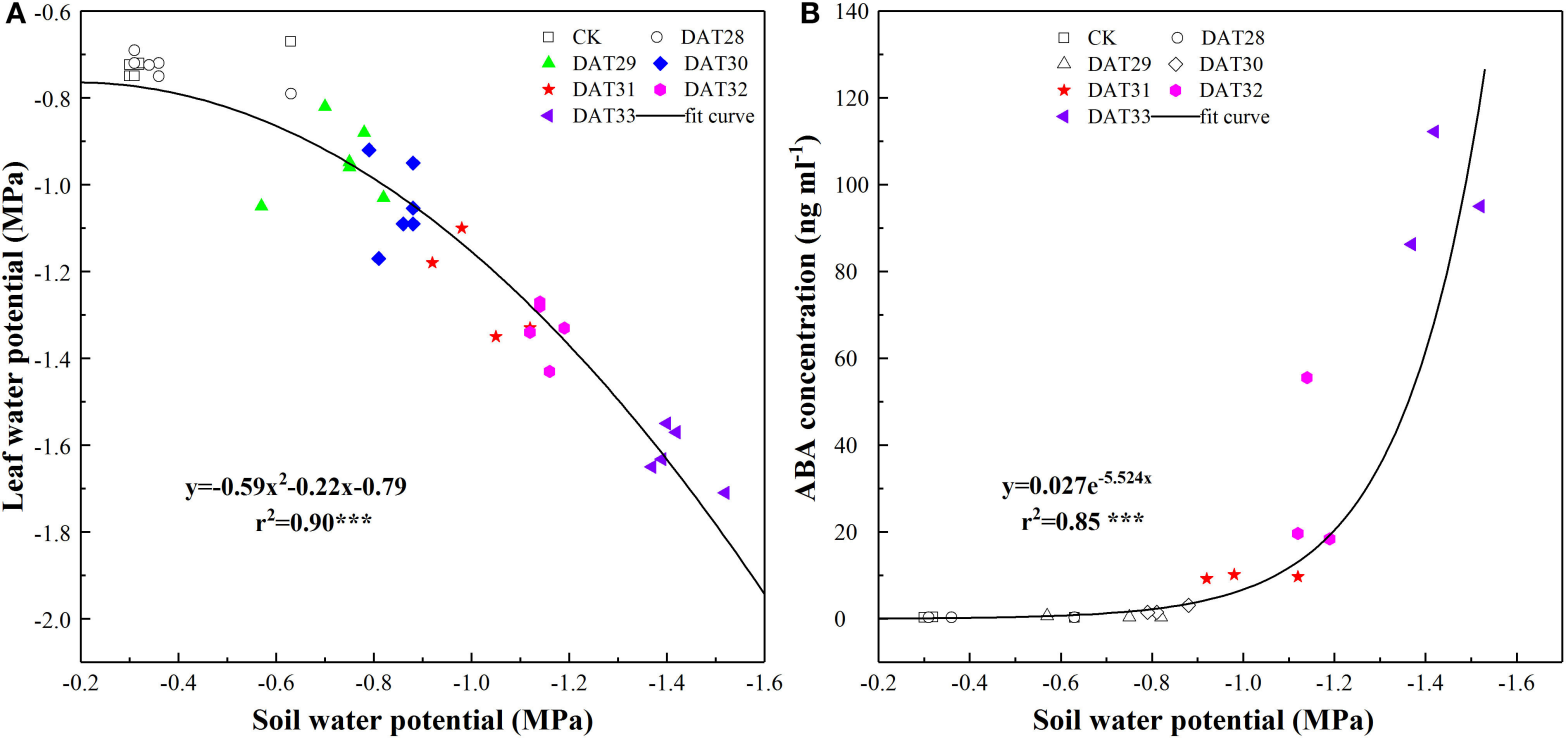
Leaf water potential (*n* = 6) **(A)** and shoot sap ABA concentration (*n* = 3) **(B)** in response to progressive soil water potential decrease. Colorful labels indicated significant difference at *P* < 0.001 level between well-watered and drought treatment.

In the well-watered treatment, Ψ_leaf_ maintained at an average of −0.72 MPa from 27 to 33 DAT. Along with decreasing Ψ_soil_ in the pots, Ψ_leaf_ of the drought-stressed tomato seedlings kept almost constant until Ψ_soil_ reached to −0.71 MPa (Figure 2A). However, ABA did not statistically increase within the range of Ψ_soil_ from −0.42 to −0.83 MPa, indicating that compared to Ψ_leaf_, chemical signal, ABA showed a delayed response in face to mild soil drying. As soil further drying, ABA increased exponentially with Ψ_soil_ decreasing from −1.01 to −1.44 MPa (Figure 2B). It should be noteworthy that ABA in the drought-stressed plants increased up to an average of 97.86 ng ml^−1^ at the end of experiment, resulting in an around 300 times higher than the well-watered treatment.

### Quantitative Analysis of Photosynthetic Limitation in Response to Soil Drying

**Figure 3:**
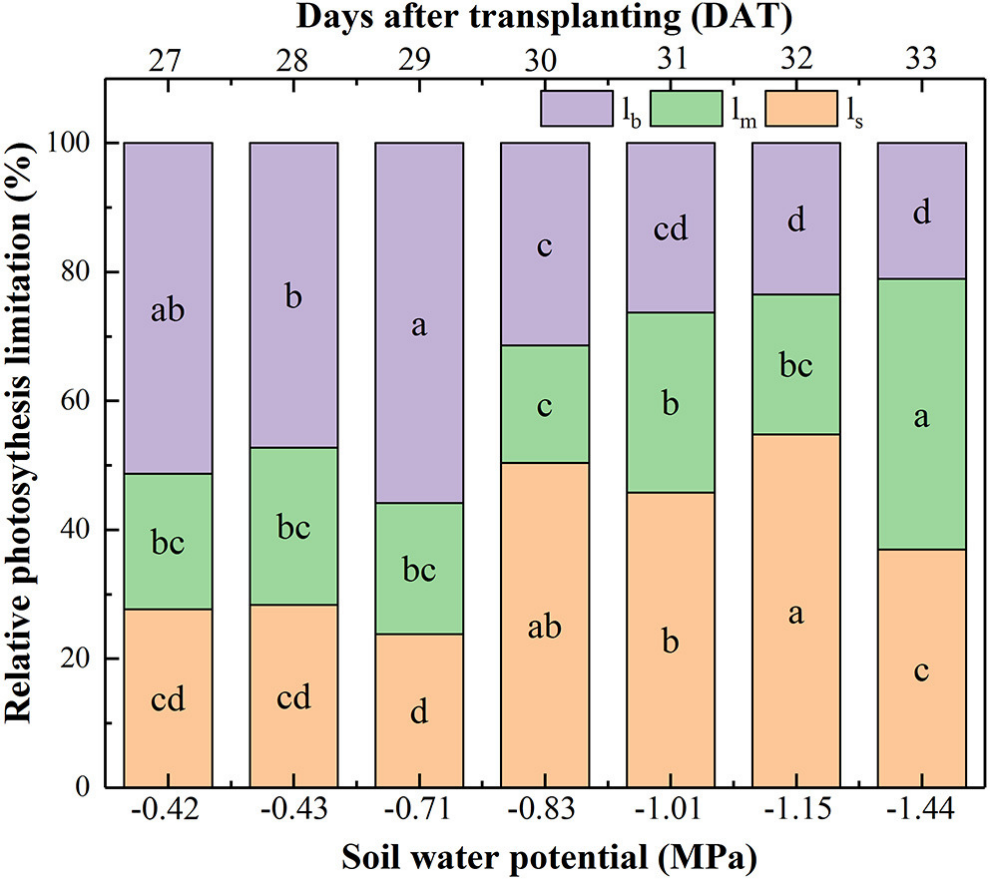
Effect of soil water potential (Ψ_soil_) on the relative contribution of the photosynthesis capacity limiting factors: limitations of A_n_ resulting from g_s_ (l_s_), g_m_ (l_m_), and biochemical photosynthetic capacity (l_b_) after transplanting. Data were means. Different letters indicated statistically significant difference between well-watered (CK) and drought plants at *P* < 0.05 level.

The relative contributions of all limiting factors (l_s_, l_m_, l_b_) to photosynthetic capacity can be divided into three stages (Figure 3). Firstly, l_b_ contributed to around an average of 51.46% limitation when Ψ_soil_ was >-0.71 MPa, suggesting that photosynthetic biochemistry was the main factor under no water stressed condition. Secondly, with Ψ_soil_ decreasing from −0.83 to −1.15 MPa, l_b_ declined, whereas both l_s_ and l_m_ increased, but l_s_ was higher than l_m_, which contributed solely to an almost 50.30% reduction in A_n_, indicating that g_s_ was the main limiting factor to photosynthetic capacity under mild and moderate drought. Thirdly, with Ψ_soil_ decreasing to −1.44 MPa, l_m_ contributed to 41.99% reduction in photosynthesis, followed by l_s_ (36.93%) and l_b_ (21.08%), showing that g_m_ was the most important limiting factor to photosynthetic capacity under the severe drought condition.

### Ψ_leaf_ and ABA in the Regulation of g_s_, g_m_, g_t_, and A_n_

**Figure 4:**
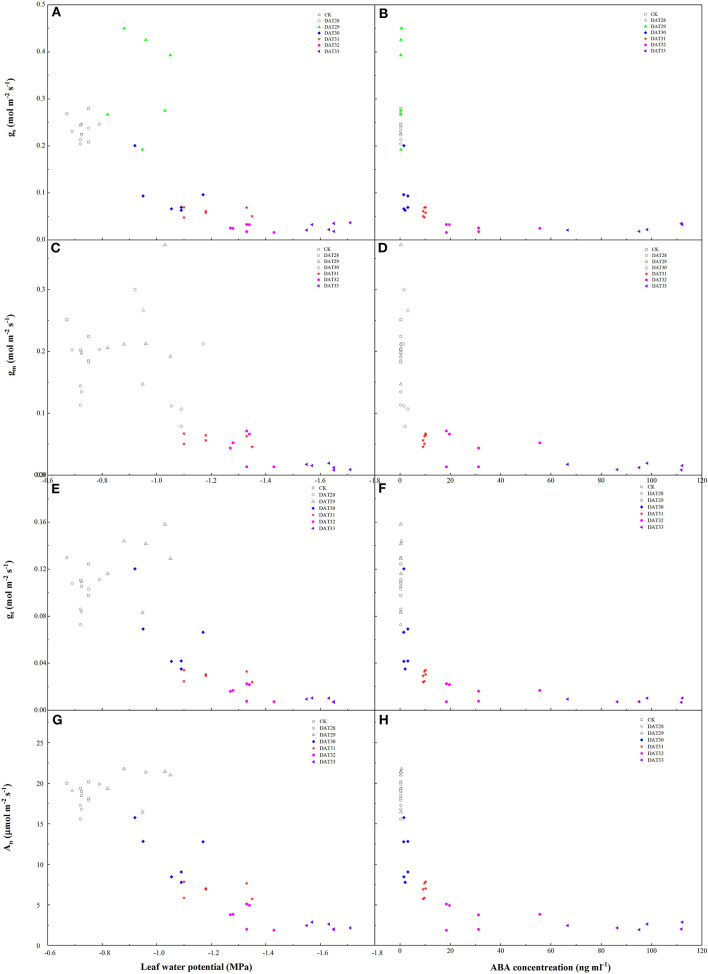
Effects of leaf water potential, ABA concentration on stomatal conductance (g_s_) **(A,B)**, mesophyll conductance (g_m_) **(C,D)**, total conductance (g_t_) **(E,F)**, and net photosynthesis (A_n_) **(G,H)**. Colorful labels indicated significant difference between the well-watered (CK) and drought treatments at *P* < 0.01 level.

**Figure 5:**
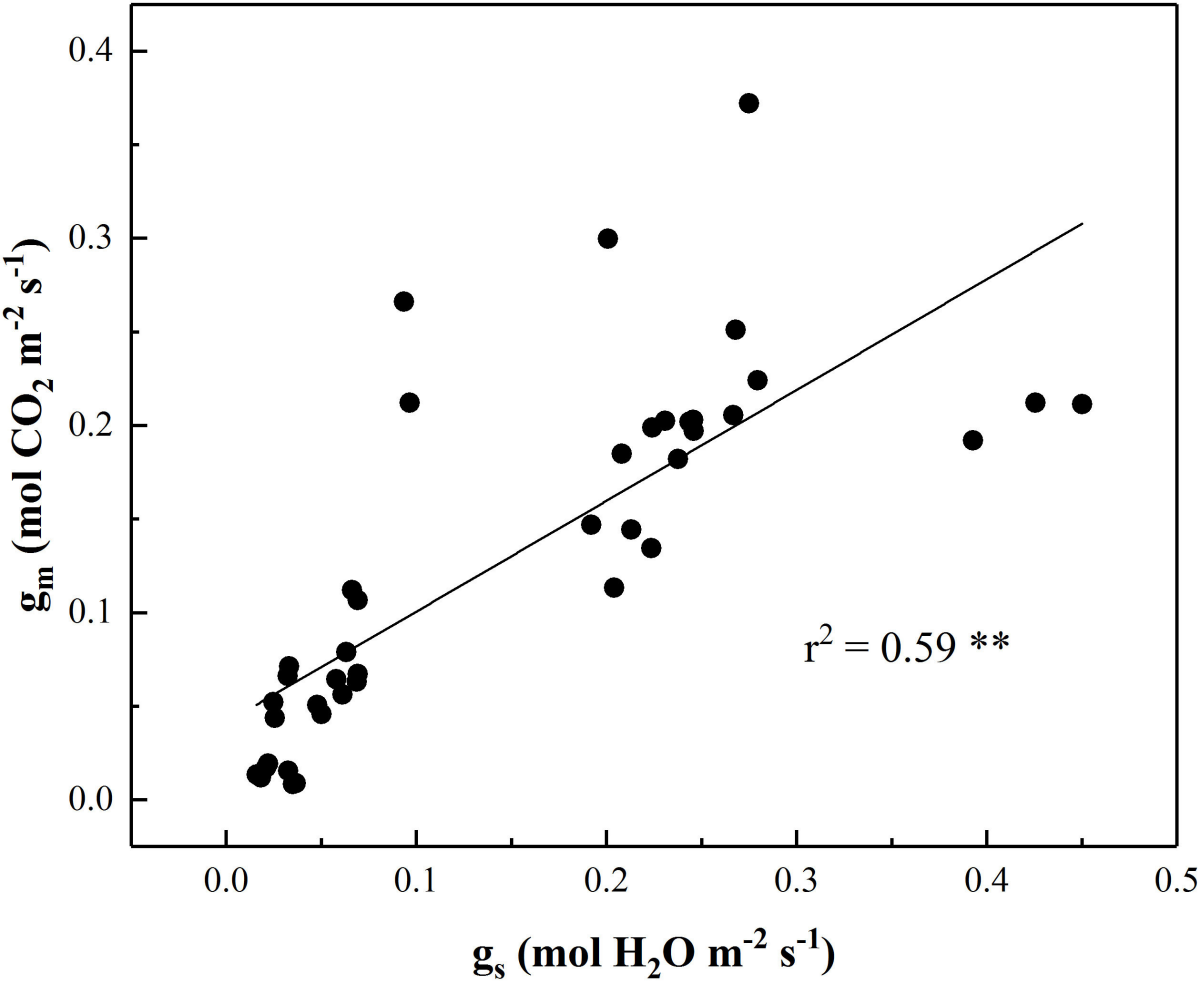
The relationship between the stomatal conductance to H_2_O (g_s_, mol H_2_O m^−2^ s^−1^) and mesophyll conductance to CO_2_ (g_m_, mol CO_2_ m^−2^ s^−1^) in the leaves under progressive drought. Data were fitted by a linear regression with *r*^2^ = 0.59 at *P* < 0.01 level.

As compared to g_s_ in CK, g_s_ in the water-stressed tomato seedlings increased firstly with Ψ_leaf_ decreasing from −0.72 to −0.95 MPa and then decreased with Ψ_leaf_ decreasing from −1.05 to −1.63 MPa (Figure 4A). However, g_m_ kept unchanged within the range of Ψ_leaf_ from −0.72 to −1.05 MPa and decreased significantly when Ψ_leaf_ was <-1.28 MPa (Figure 4C). The output of ANOVA showed that drought had significant effect on the slopes of the regression lines between g_s_ and g_m_ to Ψ_leaf_ (Supplementary Figure 1). In addition, under mild and moderate drought, the ratio of g_s_ reduction was higher than g_m_ during 30–32 DAT (Supplementary Figure 2). These results indicated that g_s_ was more sensitive to mild and moderate drought stress than g_m_. In summary, there was a significant positive relationship between Ψ_leaf_ and g_s_ (*r* = 0.74, *P* < 0.01) and g_m_ (*r* = 0.76, *P* < 0.01) during progressive soil drying (Table 1). We also investigated the relationship between ABA and g_s_ or g_m_ (Figures 4B,D). g_s_ changed with no significant increasing ABA during 28–29 DAT. As soil further dried, g_s_ continued decreasing and g_m_ started to decrease with significant increase in ABA (Figures 4B,D). g_m_ was closely related to g_s_ during drying (*r*^2^=0.59, *P* < 0.01) (Figure 5). Notably, g_m_ and ABA changed concurrently at the threshold of Ψ_soil_ = −1.01 MPa (Figures 2B, 4D). In summary, ABA was negatively related to g_m_ (*r* = −0.64, *P* < 0.01) and g_s_ (*r* = −0.55, *P* < 0.01) (Table 1). These results indicated that the decline of g_s_ was regulated by Ψ_leaf_ in the early stage of drought, whereas under moderate or severe drought, g_s_ and g_m_ were controlled by both Ψ_leaf_ and ABA.

**Table 1:** Correlation matrix between studied parameters including intrinsic water use efficiency (WUE_i_), net photosynthesis (A_n_), mesophyll conductance (g_m_), stomatal conductance (g_s_) and the ratio (g_m_/g_s_), abscisic acid (ABA), and leaf water potential (Ψ_leaf_).

	**A_**n**_**	**g_**s**_**	**g_**m**_**	**g_**m**_/g_**s**_**	**WUE_**i**_**	**Ψ_leaf_**	**ABA**
**A**_**n**_	1	0.938**	0.892**	−0.164	0.639**	0.885**	−0.695**
**g**_**s**_		1	0.777**	0.339*	0.759**	0.740**	−0.548**
**g**_**m**_			1	0.160	0.439**	0.760**	−0.643**
**g**_**m**_ **/g**_**s**_				1	0.771**	−0.109	−0.229
**WUE**_**i**_					1	−0.395**	0.072
**Ψ_leaf_**						1	−0.816**
**ABA**							1

* and **mean statistically significant relationship according to the Pearson correlation analysis at P < 0.05 and P < 0.01.

Drought significantly affected A_n_ and g_t_ during 30–33 DAT. When Ψ_leaf_ decreased to −1.05 MPa or ABA increased to 2.04 ng ml^−1^, A_n_ and g_t_ declined by 40.18 and 45.13%, respectively (Figures 4E–H). As soil further dried, i.e., Ψ_leaf_ decreasing from −1.28 to −1.63 MPa, A_n_ and g_t_ reduced by 62.84–88.94% and 74.33–92.92% in the drought-stressed plants as compared with the well-watered plants, respectively (Figures 4E,G).

### g_m_/g_s_ and WUE_i_ in Response to Ψ_leaf_ and ABA Under Progressive Soil Drying

**Figure 6:**
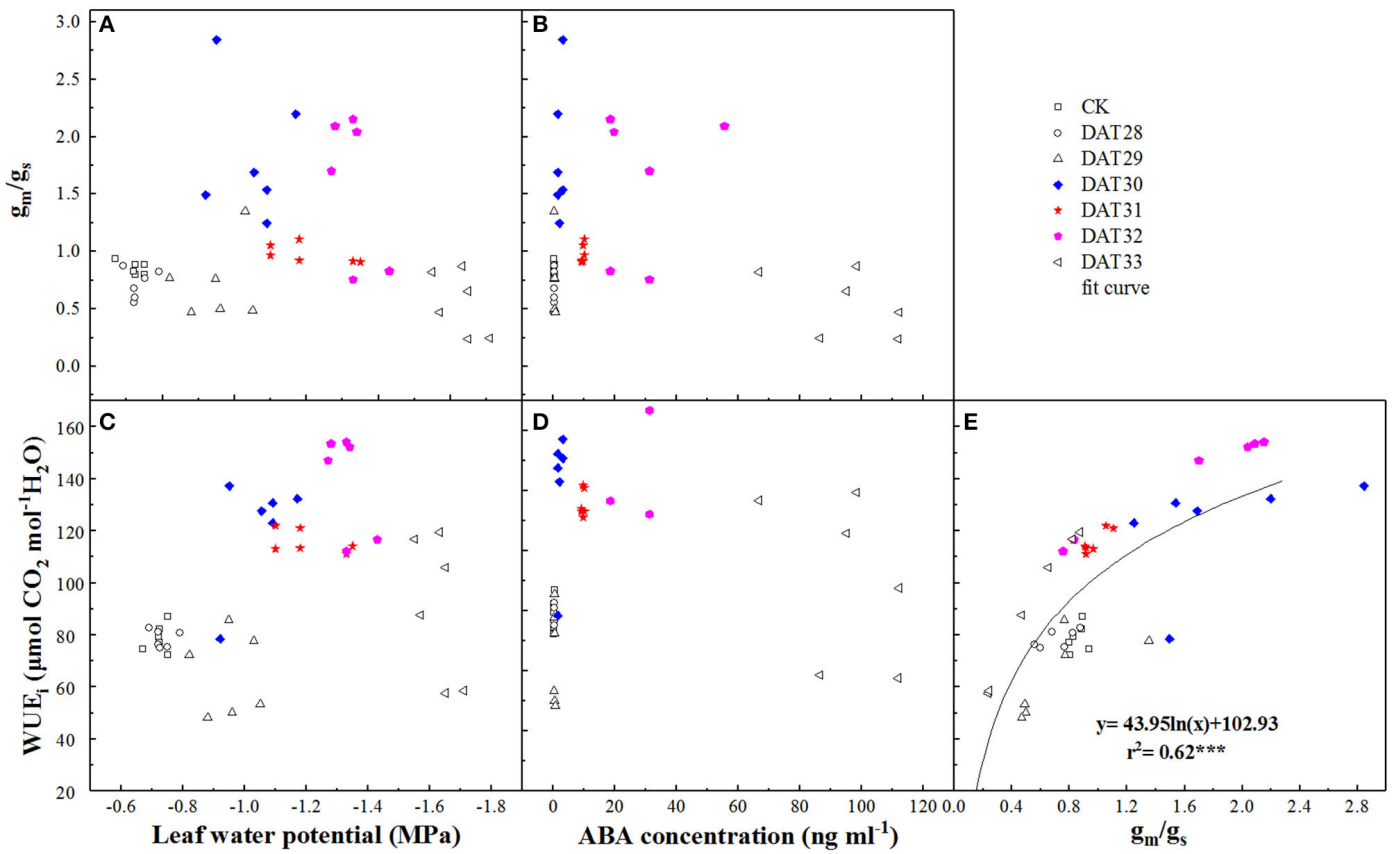
Correlation between ratio of mesophyll conductance to stomatal conductance (g_m_/g_s_) or intrinsic water use efficiency (WUE_i_) and leaf water potential **(A,C)** or shoot sap ABA **(B,D)** under progressive drought. The relationship between g_m_/g_s_ and WUE_i_ was presented with a non-linear regression at *P* < 0.001 level **(E)**. Colorful labels indicated significant difference between the well-watered (CK) and drought treatments at *P* < 0.01 level.

The dynamics of g_m_/g_s_ in response to Ψ_leaf_ and ABA during progressive soil drying were presented in Figure 6. Higher g_m_/g_s_ was observed as Ψ_leaf_ decreased from −1.05 to −1.33 MPa or as ABA increased from 2.04 to 31.23 ng ml^−1^ (Figures 6A,B), indicating that g_s_ declined more than g_m_ under mild or moderate drought. However, no significant difference of g_m_/g_s_ between CK and the intense water stress with Ψ_leaf_ = −1.63 MPa was found. WUE_i_ in response to these signals changed in the same way as g_m_/g_s_ (Figures 6C,D), it increased firstly and then decreased. In addition, WUE_i_ was positively related to g_m_/g_s_ with a logarithmic relationship (*r*^2^ = 0.62, *P* < 0.001) during the progressive soil drying (Figure 6E), indicating that WUE_i_ was strongly correlated to g_m_/g_s_.

### Sensitivity Analyses of Parameters in the Estimation g_m_

10% variation of R_d_ and J_f_ did not affect g_m_ significantly, whereas Γ^*^ has a significantly effect on g_m_ in well-watered plants (Table 2). As compared to g_m_ in the well-watered plants, g_m_ in the drought treatment was unaffected by the 20% underestimation of J_f_, showing that g_m_ in the drought treatment was less sensitive to J_f_ than in the well-watered plants. Variation of C_i_ resulted in an overestimation of g_m_ in well-watered plants, whereas g_m_ in drought treatment was unaffected by overestimation of C_i_. These results indicated that overestimation of C_i_ had a slighter effect on calculation of g_m_ than underestimation in the current study.

**Table 2:** Sensitivity analyses of the effects of ±20% error of light mitochondrial respiration (R_d_), chloroplast CO_2_ compensation point (Γ^*^), electron transport rate (*J*_f_), and intercellular CO_2_ concentration (C_i_) on calculation of g_m_ in well-watered and severe drought tomato at Ψ_soil_ = −1.44 MPa as compared with the original value of g_m_.

**Factors**	**g_**m**_ in CK**	**g_**m**_ in drought**	**Factors**	**g_**m**_ in CK**	**g_**m**_ in drought**
R_d_-20%	0.182 ± 0.006 ns	0.013 ± 0.002 ns	*J*_f_-20%	1.208 ± 0.74 **	0.014 ± 0.002 ns
R_d_-10%	0.189 ± 0.005 ns	0.013 ± 0.002 ns	*J*_f_-10%	0.309 ± 0.020 ns	0.014 ± 0.002 ns
R_d_+10%	0.206 ± 0.07 ns	0.014 ± 0.002 ns	*J*_f_+10%	0.160 ± 0.005 ns	0.013 ± 0.002 ns
R_d_+20%	0.216 ± 0.08 ns	0.014 ± 0.002 ns	*J*_f_+20%	0.141 ± 0.004 ns	0.013 ± 0.002 ns
Γ *-20%	0.146 ± 0.005 **	0.013 ± 0.002 ns	C_i_-20%	0.433 ± 0.025 **	0.020 ± 0.003 *
Γ *-10%	0.168 ± 0.009 **	0.013 ± 0.002 ns	C_i_-10%	0.270 ± 0.011 **	0.017 ± 0.003 ns
Γ ^*^+10%	0.238 ± 0.015 **	0.014 ± 0.002 ns	C_i_+10%	0.155 ± 0.005 **	0.013 ± 0.002 ns
Γ ^*^+20%	0.301 ± 0.011 **	0.014 ± 0.002 ns	C_i_+20%	0.127 ± 0.004 **	0.011 ± 0.002 ns

Data were mean ± SD (n = 6). ns indicated no significant difference and ^**^ indicated significant difference at P < 0.01 level between drought and well-watered treatment.

## Discussion

### Effects of g_s_ and g_m_ on A_n_ Under Soil Drought

Efficient CO_2_ fixation is important for plant acclimation to environmental factors. In the present study, the total diffusion conductance of CO_2_ (g_t_) and A_n_ declined synchronously under drought (Figures 4E–H). The total diffusion conductance of CO_2_ mainly includes g_s_ and g_m_ (Grassi and Magnani, 2005). Many authors have reported that CO_2_ diffusion from sub-stomatal cavities to chloroplasts is a significant factor determining photosynthetic capacity in C_3_ plants such as tomato (Han et al., 2016; Du et al., 2019; Xu et al., 2019). Our analysis showed that l_s_ and l_m_ increased as soil drying proceeded and contributed to an almost 68–78% reduction in A_n_ when Ψ_soil_ was <-0.83 MPa (Figure 3). Our results, as well as those of previous studies (Niinemets et al., 2009; Wang et al., 2018), confirmed the significance of g_s_ and g_m_ on assimilation rate under various drought conditions. It should be acknowledged that, many authors have highlighted the effects of leaf anatomical traits on g_m_, such as cell thickness, cell packing and area of chloroplasts exposed to the intercellular air spaces (S_c_/S) across many species including tomato (Tomas et al., 2013; Muir et al., 2014). This effect was a result of plants acclimation to the long-term stressed environmental factors lasting for weeks. However, rapid response of g_m_ to stress could occur within minutes response to elevating CO_2_ (Mizokami et al., 2018) or hours response to application of ABA (Sorrentino et al., 2016). Perhaps this meant that different mechanisms of g_m_ determination existed under short and long term drought conditions. Therefore, to minimize the effects of leaf anatomy on g_m_, we focused on the responses of g_m_ to drought stress and the involvement of ABA in a short water stress cycle.

### Response of g_s_ to Ψ_leaf_ and ABA Under Soil Drought

We found that g_s_ generally decreased as Ψ_leaf_ decreased (Figures 2A, 4A), suggesting that Ψ_leaf_ might induce stomatal closure at the early stage of drought. The mechanisms of this hydraulic regulation remain unclear, but the reduction in Ψ_leaf_ has been tightly associated with decreasing leaf hydraulic signals (leaf turgor or K_leaf_) in understanding the closure of stomata (Ripullone et al., 2007; Wang et al., 2018). On the one hand, evidences have suggested that decline of leaf turgor could explain the decrease in g_s_ within no change of ABA (Rodriguez-Dominguez et al., 2016; Huber et al., 2019), possibly due to the decrease of elastic modulus and the activity of anion channel in guard cell during leaf dehydration (Ache et al., 2010; Saito and Terashima, 2010). On the other hand, progressive drop of plant water potential might decrease xylem pressure and increase the likelihood of embolism and hydraulic failure (Martorell et al., 2014; Tombesi et al., 2015). Responding to the future unpredictable soil water availability, stomata closed to prevent water loss and avoid xylem cavitation. Here, the increase of shoot sap ABA concentration was statistically insignificant, which implied that stomatal closure was not initiated by ABA with Ψ_soil_ not approaching to −1.01 MPa (Figures 2B, 4B). Indeed, the delayed increase in leaf ABA in the present study was consistent with the recent findings that leaf ABA did not increase until after stomata closed, which was different from the actions of leaf turgor subjected to drought stress (Huber et al., 2019). However, as soil drought proceeded, g_s_ continued decreasing with significant changes in both ABA and Ψ_leaf_, suggesting that Ψ_leaf_ was not solely controlling g_s_, but chemical ABA was also involved in the reduction of g_s_. A similar variation between ABA and g_s_ was also reported by Tombesi et al. (2015), who indicated that ABA played a crucial role in maintaining stomatal closure under long and severe drought. However, it should be noteworthy that our data need to be further interpreted, as shoot sap ABA was collected in the pressurized stem and leaf tissues instead of in localized guard cells.

### Response of g_m_ to Ψ_leaf_ and ABA Under Soil Drought

The variable J method (Harley et al., 1992), as the most commonly and easily accessible approach, was used to determine g_m_ during the dry-down stage. To obtain precise calculation of g_m_, the highest possible accuracy of gas exchange and chlorophyll fluorescence were required during the process of measurement. As reported previously, the decrease in g_m_ under drought was likely to associate with an overestimation of C_i_ due to stomatal closure (Pons et al., 2009). However, the sensitivity analyses showed that an overestimation of C_i_ did not induce g_m_ decline in drought-stressed plants (Table 2). Thus, overestimation of C_i_ was unlikely to have a significant effect on g_m_ in this study, might due to the influence of other environmental variations was ruled out under controlled environment. Therefore, it is reasonable to conclude that the reduction in g_m_ during drought was mostly attributed to the decline of g_m_ *per se* rather than the overestimation of C_i_.

Compared to the response of g_s_, g_m_ in the drought-stressed seedlings remained almost constant with Ψ_leaf_ not decrease to −1.28 MPa (Figures 4A,C), indicating that g_m_ was less sensitive to the decrease in Ψ_leaf_ than g_s_ at the beginning of soil drought. This result was in agreement with an earlier study conducted by Théroux-Rancourt et al. (2014), who found that g_m_ only responded to more negative Ψ_leaf_ or more severe soil drought, e.g., Ψ_soil_ < −1.01 MPa in the present study. Hydraulic compartmentalization of the mesophyll cell from the transpiration stream may account for this delayed response of g_m_ to Ψ_leaf_ (Zwieniecki et al., 2007; Théroux-Rancourt et al., 2014). This delayed response of g_m_ under the mild soil drought might be beneficial for mesophyll cells to be buffered against little variation in leaf water status and allow plants to maintain a greater A_n_ (Figure 4G).

However, as soil drought proceeded, g_m_ declined as Ψ_leaf_ continued decreasing (Figures 4C,D). Based on literature surveys, the causes of this decrease in g_m_ may be influenced by three main factors: mesophyll structure, membrane permeability, and biochemical enzymes activity (Flexas et al., 2008; Evans et al., 2009; Sorrentino et al., 2016). Mesophyll structural properties may not be involved in this rapid reduction of g_m_ under the short-term drought. Instead, it is well-established that the K_leaf_-induced reduction in g_m_ was associated with the decrease in mesophyll density or membrane permeability under drought conditions (Aasamaa et al., 2005; Xiong et al., 2018). Water moves through leaf mesophyll tissues via apoplastic, symplastic and vapor phase pathways, which shared a part of pathways of CO_2_ diffusion (Xiong and Nadal, 2020). The decline in hydraulic conductance under drought usually leads to reductions in water supply to the leaves, therefore affecting mesophyll cells water relations and functions. Although the effect of K_leaf_ on g_m_ was not investigated in this study, we observed a strong and positive relationship between Ψ_leaf_ and g_m_ (*r*^2^ = 0.77, *P* < 0.01) (Figure 4C), because K_leaf_ was strongly influenced by Ψ_leaf_ under drought stress (Wang et al., 2018). Therefore, the decline in Ψ_leaf_ might contribute to this decrease in g_m_, as CO_2_ diffusion and liquid water shared partly common pathways within leaves (Xiong et al., 2018).

Most notably, rapid reduction of g_m_ occurred following with increase of ABA when Ψ_soil_ was below −1.01 MPa in the current study. Fast fluctuations in g_m_ have also been recorded in response to ABA application (Sorrentino et al., 2016; Mizokami et al., 2018). The concurrent responses between g_m_ and ABA with Ψ_soil_ decreasing from −1.01 to −1.44 MPa was not a mere coincidence. This might suggest that Ψ_leaf_ was not the only factor influencing g_s_ and g_m_ under drought, other signals (ABA) could be involved in this reduction. Though mechanisms for the effect of ABA on g_m_ remain unclear, the results from both Sorrentino et al. (2016) and the current studies indicated that the reduction in g_m_ was most likely regulated by biochemical components due to the rapid reduction of g_m_ to ABA (Flexas et al., 2008; Kaldenhoff et al., 2008; Xiong et al., 2018). Evidences have indicated two candidates are likely to play this biochemical role: carbonic anhydrase and aquaporins. CO_2_ molecules passing from sub-stomatal cavities to chloroplasts diffuse through the gas phase among intercellular air spaces and the liquid phase from the cell wall to stroma. Carbonic anhydrase (CA) plays a key role on the conversion of gaseous CO_2_ to aqueous carbonic acid (H_2_CO_3_) (Flexas et al., 2008). Higher ABA accumulation was likely to change the extracellular pH and decrease the activity of H^+^-ATP-ase, an important ion transporter in plant cell plasma membrane, thus affect the CA activity (Hayat et al., 2001; Sukhov et al., 2017). Aquaporins (AQPs) are pore-forming integral membrane proteins that transport of water, CO_2_ and other small neutral molecules across the plasma membrane (Flexas et al., 2006; Kaldenhoff, 2012). A higher abundance of AQPs increased the cellular CO_2_ uptake rates several folds. Expressions of plant AQPs could be influenced by drought stress and ABA (Kapilan et al., 2018). Additionally, an indirect role of ABA on decreasing K_leaf_ might also be involved in regulating g_m_, due to the ability of ABA on inactivation bundle sheath aquaporins such as the plasma membrane intrinsic proteins (PIPs) (Shatil-Cohen et al., 2011; Pantin et al., 2013). Based on these, we considered that the reduction in g_m_ was not attributed solely to hydraulic regulation, ABA seemed to maintain the decrease in g_m_ under moderate or severe soil drought, e.g., Ψ_soil_ < −1.01 MPa in the present study. The regulation of g_m_ is complex, and regulated by many factors, including hydraulic or chemical signaling and mesophyll structure. It is still unclear the mechanism of g_m_ response to ABA under stress, further analysis of the expressions of carbonic anhydrase and cooporin protein in membrane may elucidate the biochemical mechanisms underlying this response. Notably, g_s_ and g_m_ decreased as ABA significantly increased (Figures 4B,D). Pooling all the data, a strong and positive relationship between both variables was observed in Table 1. In addition, 59% of the variation in g_m_ can be explained by g_s_ (Figure 5). Coupled changes between g_s_ and g_m_ was also found in response to drought (Perez-Martin et al., 2009; Han et al., 2016; Olsovska et al., 2016) or ABA application (Mizokami et al., 2018). Therefore, it seems that drought regulated g_m_ in order to match the variation of g_s_, thereby optimization balance between CO_2_ uptake and water loss. However, the role of g_s_ on regulating g_m_ response to ABA is still debated by many scientists (Sorrentino et al., 2016; Mizokami et al., 2018), further detail investigations are needed to address this issue.

### Variability of WUE_i_ Under Drought Depends on g_m_/g_s_

In this study, g_m_/g_s_ and WUE_i_ increased concurrently with Ψ_soil_ in the range of −0.83 to −1.15 MPa with a strong correlation (Figure 6E). Our results showed that WUE_i_ was closely related to g_m_/g_s_ compared to the correlation between WUE_i_ and g_m_ or g_s_ (Table 1). This result was consistent with Han et al. (2016) who also found WUE_i_ and g_m_/g_s_ were closely correlated compared to the correlation between WUE_i_ and g_s_ or g_m_. These suggested that variations in WUE_i_ were much more sensitive to changes of g_m_/g_s_. Stomata controls the water loss and mesophyll determines the photosynthesis, thus it would be better that using g_m_/g_s_ instead of A_n_/g_s_ explained the variations of WUE_i_. Interestingly, this improvement of WUE_i_ were coupled with increase in ABA. This might due to g_s_ reduced more in response to ABA than g_m_ under moderate drought. Though the mechanisms of ABA improving WUE_i_ remain largely unknown, it is likely to be one of the most promising strategies to improve WUE_i_ by means of decoding of the ABA signaling pathway or manipulating the expression of ABA-related genes on stomatal conductance or CA activity (Flexas et al., 2016; Cardoso et al., 2020). Nonetheless, such improvement of WUE_i_ controlled by ABA could only be beneficial for maintaining water status under short-term drought during Ψ_soil_ reduction from −0.83 to −1.15 MPa, not for long and serious drought (Figure 6D). This was beacuse the increase in WUE_i_ at leaf scale may not always mean an improvement of WUE at the whole plant scale under serious soil drought, as the closure of stomata restricts CO_2_ uptake and hence diminish plant productivity (Xue et al., 2016).

## Conclusion

The limitation of g_s_ and g_m_ increased along with progressive soil drying and diffusive conductance to CO_2_ from ambient air to chloroplasts was the crucial constraints to photosynthesis under drought conditions. The decrease in Ψ_leaf_ triggered stomata closure at the onset of drought. As soil drying proceeded, g_s_ and g_m_ declined synchronously. Both hydraulic and ABA signals were involved in this consistent decrease under moderate and severe drought. WUE_i_ improved as g_m_/g_s_ increased under mild and moderated drought due to a larger reduction of g_s_ to ABA than g_m_. Manipulation of ABA levels might be a promising approach to improve plant water use efficiency for breeding project. For future research, examining the influence of stomatal closure on g_m_ response to ABA will give further detailed insight on working of g_m_ to ABA.

## Data Availability Statement

The original contributions presented in the study are included in the article/Supplementary Material, further inquiries can be directed to the corresponding authors.

## Author Contributions

AD and YG planned and designed the experiments. SL and JL performed the experiments and analyzed the data. SL wrote the draft manuscript. AD, YG, HL, and RQ revised the manuscript. All authors read and approved the final manuscript.

## Conflict of Interest

The authors declare that the research was conducted in the absence of any commercial or financial relationships that could be construed as a potential conflict of interest.

## Supplementary Material

Supplementary Figure 1 Relationships between stomatal conductance (g_s_) or mesophyll conductance (g_m_) under drought during 30-33 DAT. Closed circles indicated g_s_, open circles indicated g_m_. Slope with P value indicates significant difference between the slopes of the regression lines for g_s_ and g_m_ to Ψ_leaf_.

Supplementary Figure 2 The decreasing ratio of stomatal conductance (g_s_) or mesophyll conductance (g_m_) under drought compared CK during 30 to 33 DAT.
