# Corrigendum: Role of Hydraulic Signal and ABA in Decrease of Leaf Stomatal and Mesophyll Conductance in Soil Drought-Stressed Tomato

**DOI:** 10.3389/fpls.2021.710792

**Published:** 2021-08-13

**Authors:** Shuang Li, Junming Liu, Hao Liu, Rangjian Qiu, Yang Gao, Aiwang Duan

**Affiliations:** ^1^Key Laboratory of Crop Water Use and Regulation, Ministry of Agriculture and Rural Affairs, Farmland Irrigation Research Institute, Chinese Academy of Agricultural Sciences, Xinxiang, China; ^2^Graduate School of Chinese Academy of Agricultural Sciences, Beijing, China; ^3^School of Applied Meteorology, Nanjing University of Information Science and Technology, Nanjing, China

**Keywords:** drought, leaf water potential, abscisic acid, stomatal conductance, mesophyll conductance, intrinsic water use efficiency

In the original article, there was an error in Figure 1 as published. The value of Ψ_soil_ at 33 DAT should be −1.44 MPa. The corrected Figure 1 appears here.

**Figure 1:**
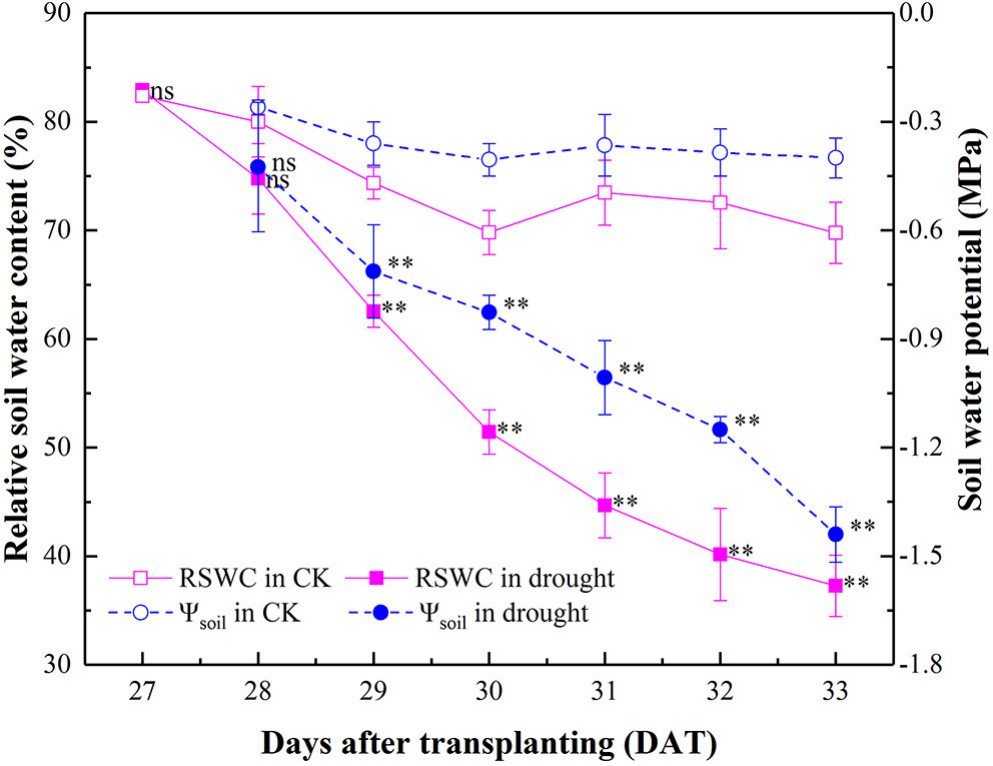
Dynamics of RSWC and Ψ_soil_ in the well-watered (CK) and drought-stressed tomato seedlings during 27–33 DAT. Mean values and SD were presented (*n* = 6). ns indicated no significant difference and ^**^ indicated significant difference at *P* < 0.01 level between drought and well-watered treatment.

The associated text in the *Results* section *Dynamic of Soil Water Status* has also been updated to reflect the correction to Figure 1, as described below.

The originally published sentence “By withholding irrigation from 27 to 33 DAT during the progressive drying process, RSWC in the drought treatment decreased gradually from 82.90 to 37.27% and Ψ_soil_ decreased by 1.12 MPa correspondingly.” has been corrected to read “By withholding irrigation from 27 to 33 DAT during the progressive drying process, RSWC in the drought treatment decreased gradually from 82.90 to 37.27% and Ψ_soil_ decreased by 1.04 MPa correspondingly.”

In the original article, there was an error in Figure 3 as published. The value of Ψ_soil_ at 33 DAT should be −1.44 MPa. The corrected Figure 3 appears here.

**Figure 3:**
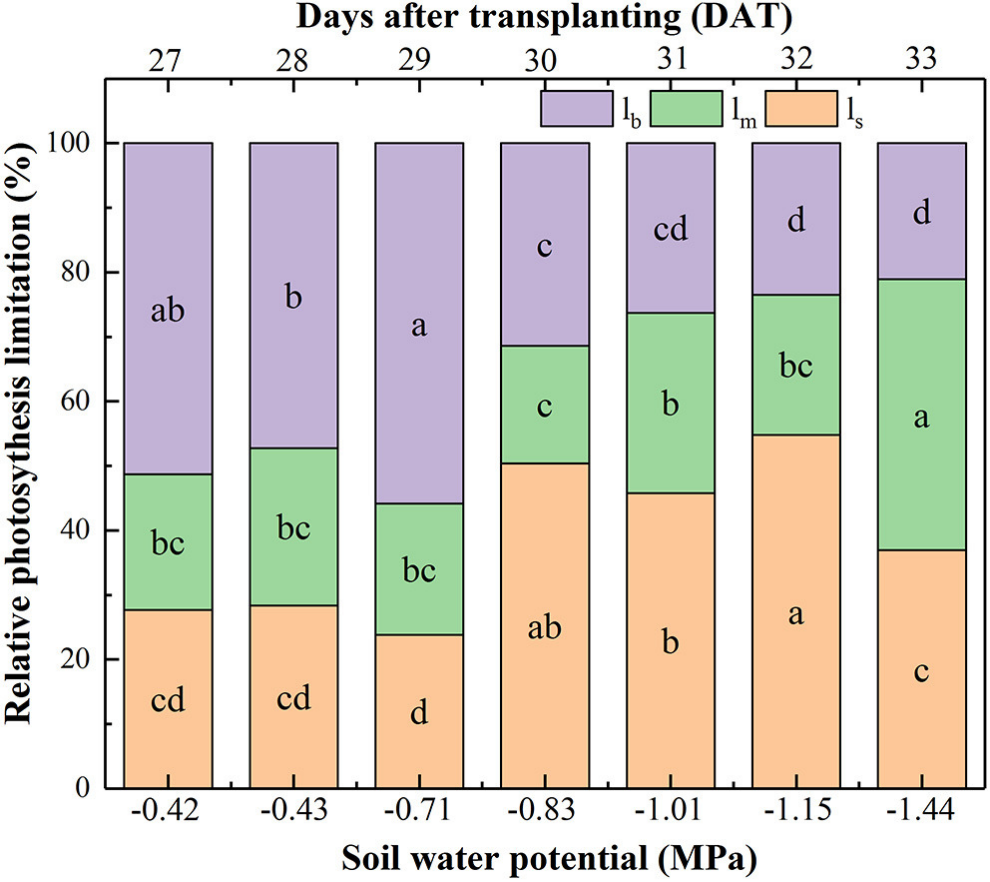
Effect of soil water potential (Ψ_soil_) on the relative contribution of the photosynthesis capacity limiting factors: limitations of A_n_ resulting from g_s_ (l_s_), g_m_ (l_m_), and biochemical photosynthetic capacity (l_b_) after transplanting. Data were means. Different letters indicated statistically significant difference between well-watered (CK) and drought plants at *P* < 0.05 level.

The associated text in the *Results* section *Quantitative Analysis of Photosynthetic Limitation in Response to Soil Drying* has also been updated to reflect the correction to Figure 3, as described below.

The originally published sentence “Thirdly, with Ψ_soil_ decreasing to −1.54 MPa, l_m_ contributed to 41.99% reduction in photosynthesis, followed by l_s_ (36.93%) and l_b_ (21.08%), showing that g_m_ was the most important limiting factor to photosynthetic capacity under the severe drought condition.” has been corrected to read “Thirdly, with Ψ_soil_ decreasing to −1.44 MPa, l_m_ contributed to 41.99% reduction in photosynthesis, followed by l_s_ (36.93%) and l_b_ (21.08%), showing that g_m_ was the most important limiting factor to photosynthetic capacity under the severe drought condition.”

In the original article, there were errors in Table 2 as published. Owing to a miscalculation, the values of the parameters were incorrect. The corrected Table 2 appears here.

**Table 2:** Sensitivity analyses of the effects of ±20% error of light mitochondrial respiration (R_d_), chloroplast CO_2_ compensation point (Γ^*^), electron transport rate (*J*_f_), and intercellular CO_2_ concentration (C_i_) on calculation of g_m_ in well-watered and severe drought tomato at Ψ_soil_ = −1.44 MPa as compared with the original value of g_m_.

**Factors**	**g_**m**_ in CK**	**g_**m**_ in drought**	**Factors**	**g_**m**_ in CK**	**g_**m**_ in drought**
R_d_-20%	0.182 ± 0.006 ns	0.013 ± 0.002 ns	*J*_f_-20%	1.208 ± 0.74 **	0.014 ± 0.002 ns
R_d_-10%	0.189 ± 0.005 ns	0.013 ± 0.002 ns	*J*_f_-10%	0.309 ± 0.020 ns	0.014 ± 0.002 ns
R_d_+10%	0.206 ± 0.07 ns	0.014 ± 0.002 ns	*J*_f_+10%	0.160 ± 0.005 ns	0.013 ± 0.002 ns
R_d_+20%	0.216 ± 0.08 ns	0.014 ± 0.002 ns	*J*_f_+20%	0.141 ± 0.004 ns	0.013 ± 0.002 ns
Γ *-20%	0.146 ± 0.005 **	0.013 ± 0.002 ns	C_i_-20%	0.433 ± 0.025 **	0.020 ± 0.003 *
Γ *-10%	0.168 ± 0.009 **	0.013 ± 0.002 ns	C_i_-10%	0.270 ± 0.011 **	0.017 ± 0.003 ns
Γ ^*^+10%	0.238 ± 0.015 **	0.014 ± 0.002 ns	C_i_+10%	0.155 ± 0.005 **	0.013 ± 0.002 ns
Γ ^*^+20%	0.301 ± 0.011 **	0.014 ± 0.002 ns	C_i_+20%	0.127 ± 0.004 **	0.011 ± 0.002 ns

Data were mean ± SD (n = 6). ns indicated no significant difference and ^**^ indicated significant difference at P < 0.01 level between drought and well-watered treatment.

The associated text has also been updated to reflect to reflect the correction to Table 2, as described below.

In the *Results* section *Sensitivity Analyses of Parameters in the Estimation g*_*m*_, the originally published sentence “20% variation of R_d_, Γ^*^ did not affect g_m_ significantly (Table 2).” has been corrected to read “10% variation of R_d_ and J_f_ did not affect g_m_ significantly, whereas Γ^*^ has a significantly effect on g_m_ in well-watered plants (Table 2).”

In the *Results* section *Sensitivity Analyses of Parameters in the Estimation g*_*m*_, the originally published sentence “20% underestimation of C_i_ resulted in an overestimation of g_m_, while g_m_ was unaffected by overestimation of C_i_ in both the well-watered and drought treatments.” has been corrected to read “Variation of C_i_ resulted in an overestimation of g_m_ in well-watered plants, whereas g_m_ in drought treatment was unaffected by overestimation of C_i_.”

In the *Discussion* section *Response of g*_*m*_ *to* Ψ_*leaf*_ *and ABA Under Soil Drought*, the originally published sentence “However, the sensitivity analyses showed that an overestimation of C_i_ did not induce g_m_ decline neither in the well-watered nor drought-stressed plants (Table 2).” has been corrected to read “However, the sensitivity analyses showed that an overestimation of C_i_ did not induce g_m_ decline in drought-stressed plants (Table 2).”

In the original article, there were errors (incorrect *P*-values) in the following sentence from the *Results* section Ψ_*leaf*_ *and ABA in the Regulation of g*_*s*_*, g*_*m*_*, g*_*t*_, and *A*_*n*_: “In summary, ABA was negatively related to g_m_ (*r* = −0.64, *P* < 0.001) and g_s_ (*r* = −0.55, *P* < 0.001) (**Table 1**).” The sentence should have read “In summary, ABA was negatively related to g_m_ (*r* = −0.64, *P* < 0.01) and g_s_ (*r* = −0.55, *P* < 0.01) (**Table 1**).”

The authors apologize for these errors and state that they do not change the scientific conclusions of the article in any way. The original article has been updated.

## Publisher's Note

All claims expressed in this article are solely those of the authors and do not necessarily represent those of their affiliated organizations, or those of the publisher, the editors and the reviewers. Any product that may be evaluated in this article, or claim that may be made by its manufacturer, is not guaranteed or endorsed by the publisher.

