# Supplementary figures and images for: Role of Hydraulic Signal and ABA in Decrease of Leaf Stomatal and Mesophyll Conductance in Soil Drought-Stressed Tomato
Source: Front Plant Sci. 2021 Apr 29;12:653186. doi: 10.3389/fpls.2021.653186 (PMC8118518; doi:10.3389/fpls.2021.653186)

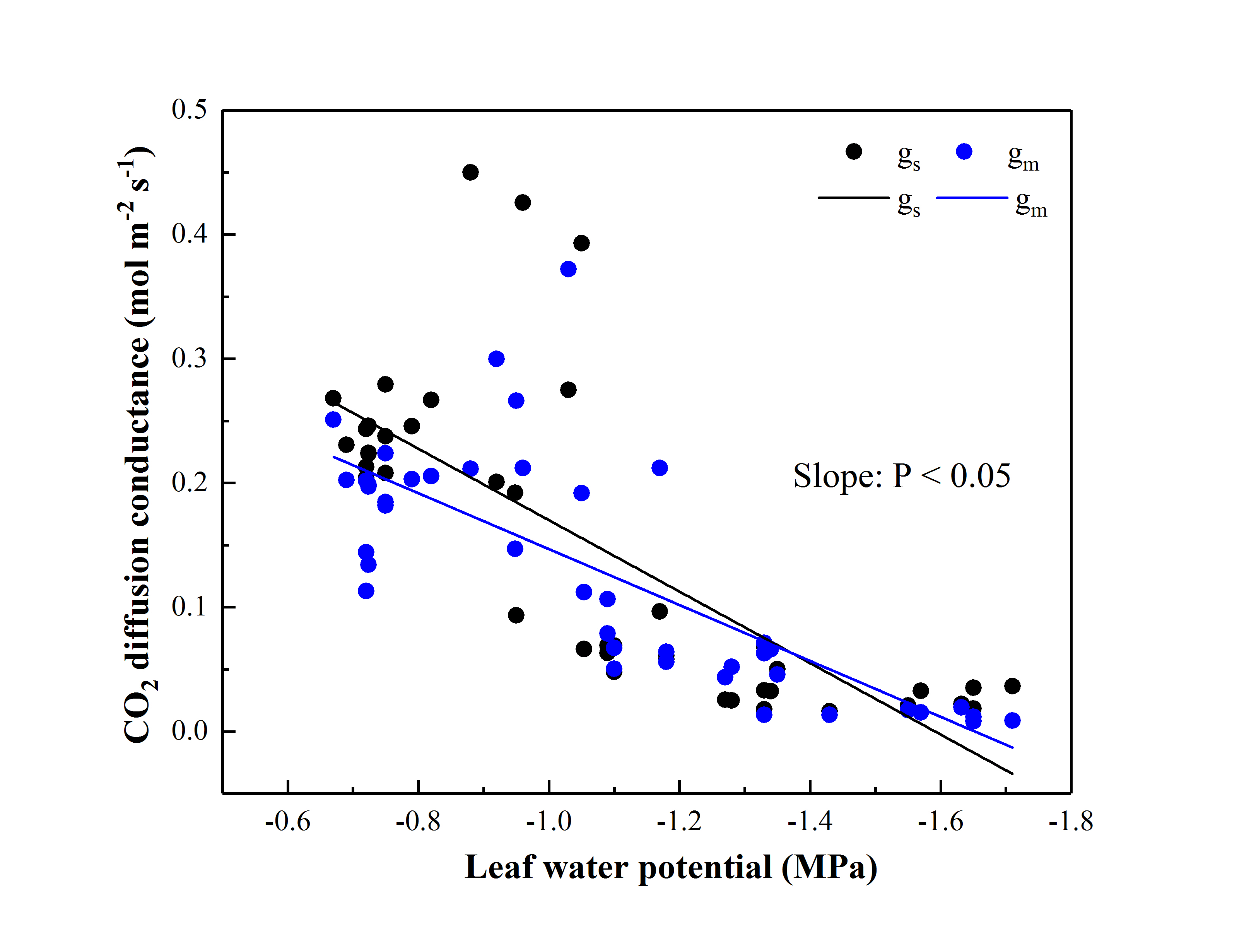

Supplement: Supplementary Figure 1 — Relationships between stomatal conductance (gs) or mesophyll conductance (gm) under drought during 30-33 DAT. Closed circles indicated gs, open circles indicated gm. Slope with P value indicates significant difference between the slopes of the regression lines for gs and gm to Ψleaf. [file Image_1.JPEG]

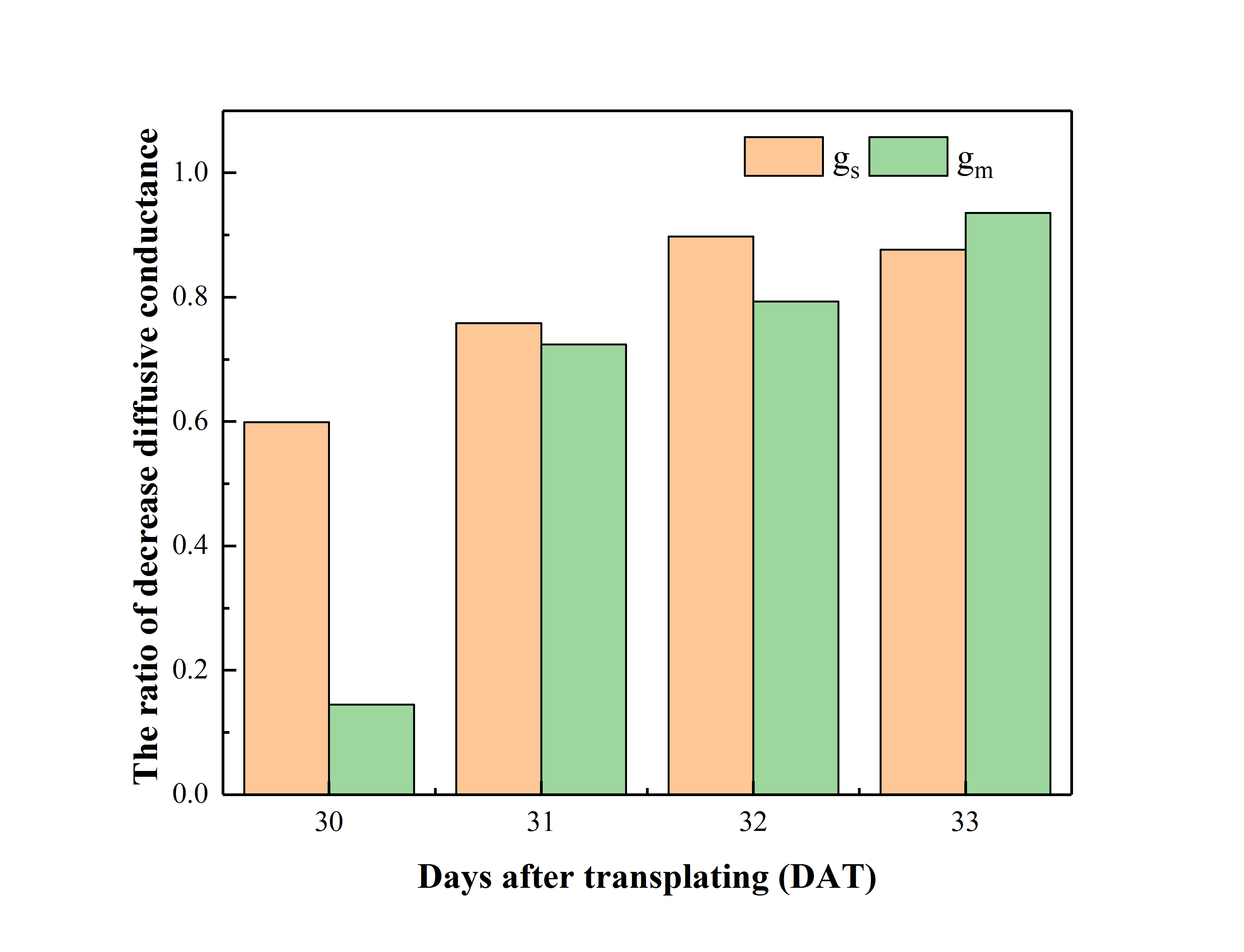

Supplement: Supplementary Figure 2 — The decreasing ratio of stomatal conductance (gs) or mesophyll conductance (gm) under drought compared CK during 30 to 33 DAT. [file Image_2.JPEG]
